# Does Computerized Physician Order Entry Benefit from Dynamic Structured Data Entry? A Quasi-Experimental Study

**DOI:** 10.1186/s12911-018-0709-4

**Published:** 2018-11-26

**Authors:** George Linn, Yung-Hsiang Ying, Koyin Chang

**Affiliations:** 1 Department of Obstetrics and Gynecology, Kaohsiung Medical University Hospital, Kaohsiung, Taiwan; 2 Department of Healthcare Information and Management, Ming Chuan University, No 5, De-Ming Rd, Taoyuan, Taiwan; 3 College of Management, National Taiwan Normal University, 162 Hoping E Rd. Sec 1, Taipei, Taiwan

**Keywords:** Computerized physician order entry, Dynamic structured data entry, Quasi-experimental study, Electronic medical record

## Abstract

**Background:**

With advancements in information technology, computerized physician order entry (CPOE) and electronic Medical Records (eMR), have become widely utilized in medical settings. The predominant mode of CPOE in Taiwan is free text entry (FTE). Dynamic structured data entry (DSDE) was introduced more recently, and has increasingly drawn attention from hospitals across Taiwan. This study assesses how DSDE compares to FTE for CPOE.

**Methods:**

A quasi-experimental study was employed to investigate the time-savings, productivity, and efficiency effects of DSDE in an outpatient setting in the gynecological department of a major hospital in Taiwan. Trained female actor patients were employed in trials of both entry methods. Data were submitted to Shapiro-Wilk and Shapiro-Francia tests to assess normality, and then to paired t-tests to assess differences between DSDE and FTE.

**Results:**

Relative to FTE, the use of DSDE resulted in an average of 97% time saved and 55% more abundant and detailed content in medical records. In addition, for each clause entry in a medical record, the time saved is 133% for DSDE compared to FTE.

**Conclusion:**

The results suggest that DSDE is a much more efficient and productive entry method for clinicians in hospital outpatient settings. Upgrading eMR systems to the DSDE format would benefit both patients and clinicians.

## Background

Medical records are essential to hospitals’ and healthcare providers’ ability to retain patient information for reference for future treatment. Medical records also constitute a critical source of data for medical research. However, the medical narrative section of the patient record, which includes medical history, progress reports, and notes on diagnoses and interventions, often varies across disciplines [1]. In addition to such cross-discipline variability, the level of detail in visit notes fluctuates broadly between clinicians. Electronic medical record (eMR) systems facilitate the exchange and dissemination of information between different care teams, and are a secure and effective tool for maintaining patients’ healthcare data and reducing medical errors [2]. Effective eMR systems should facilitate clinicians autonomous recording of patient information accurately, effectively, and rapidly [3]. The degree to which an eMR system is developed and adapted within an institution can also reflect the state of patient care monitoring as well as institutional performance. Such systems also play important roles in facilitating clinical research and auditing processes [4, 5].

The healthcare system in Taiwan began to adopt eMR approximately 20 years ago. By 2015, approximately 90% of hospitals had converted their medical record systems to completely electronic formats (paperless); approximately 30% have even achieved inter-hospital data-sharing and collaboration [6]. Free text entry (FTE) is currently the predominant eMR mode in Taiwan. This design has been employed by Taiwan’s healthcare system for a long time. The introduction of dynamic structured data entry systems (DSDE) in 2004, has prompted some hospitals to consider switching from FTE to DSDE. However, the cost of implementing DSDE is high. Institutions continue to weigh the associated costs and benefits of these two methods. In the face of escalating pressures like stricter hospital accreditation requirements, increased volume of outpatient visits, and higher expectations of quality of care, the cost of DSDE seems less substantial than it did upon its introduction some 15 years ago.

To demonstrate whether DSDE eMR systems can save physicians’ time and effort, and generate medical notes with clearer and more abundant information, the current study compared the two eMR systems, i.e. FTE and DSDE, on particularity, productivity, efficiency, and time-spent-on-medical-note. The extant research focuses primarily on comparisons between paper records and eMR [7–11]; our study thus provides novel, useful information by comparing these two different types of entry systems. More information about relevant past literature can be found in Poissant et al. [12]. The detailed research design is as follows.

## Methods and implementation

### eMR entry system

Free text entry (FTE) consists of textboxes on a screen into which physicians type their clinical notes. The installation and training costs of FTE are relatively low as just one format of FTE fits all specialties. By contrast, DSDE is an application that supports clinicians with the recording of structured data for use in both care and research [13]. It allows tailoring of a domain model to specific medical content, and content coverage can be expanded and altered without the need for technical adaptation of the software or the physical data structure [14, 15]. DSDE expands terse medical notes into full, grammatical sentences, and can even easily render those sentences into another language, e.g. French or Chinese. The built-in transcript system can transform structured phrases into a narrative eMR that is expressed in the same way as medical notes written by physicians.

**Fig. 1:**
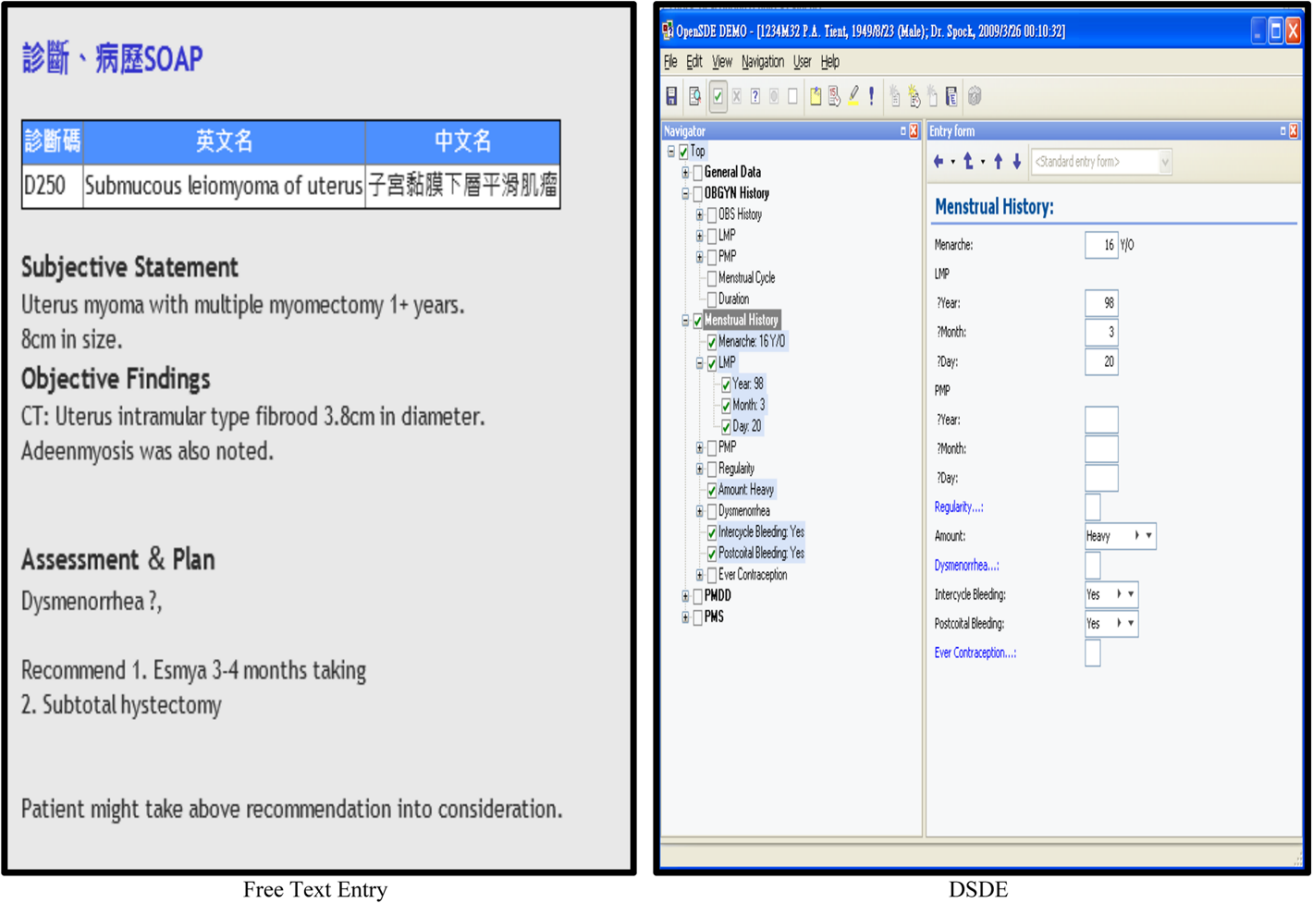
Example pictures of FTE and DSDE. Screen captures of the two interfaces. DSDE Dynamic Structured Data Entry; SOAP: Subjective, Objective, Assessment, Plan. Note: In the FTE picture, the Chinese phrases in order are “diagnosis” and “disease history” on the first line, “diagnosis code”, “English name”, and “Chinese name” on the second line, and “submucosal fibroid (or leiomyoma) of uterus” on the third line

The data entry procedures for DSDE are uniform, though content varies by specialty. Figure 1 features screen captures of both the FTE (left) and DSDE (right) software interfaces. The DSDE images show the hierarchical character of the domain models (which reflect the nature of medical descriptions). Looking at the DSDE entry screen, one sees that the left-hand side of the image shows a “tree” with predefined medical concepts. In this example, ‘Menstrual History’ is the parent concept. The right-hand side of the screen shows a daughter form, containing all concepts in the expandable tree of ‘Menstrual History’. Specific domain models are created for each medical discipline. For this reason, the implementation of the DSDE interface requires a considerable investment of time and money. In contrast, currently predominant FTE eMR systems require a low level of programming design since one FTE system fits all specialties without any modifications [16]. Moreover, hospital information technology (IT) divisions need not discuss and negotiate the interface with end-users from different medical specialties; such consultation can constitute a major burden for hospital IT personnel. Consequently, FTE is popular in cost-conscious hospital environments [17] and many hospitals in Taiwan are reluctant to switch entry systems from FTE to DSDE.

### Experiment design

**Fig. 2:**
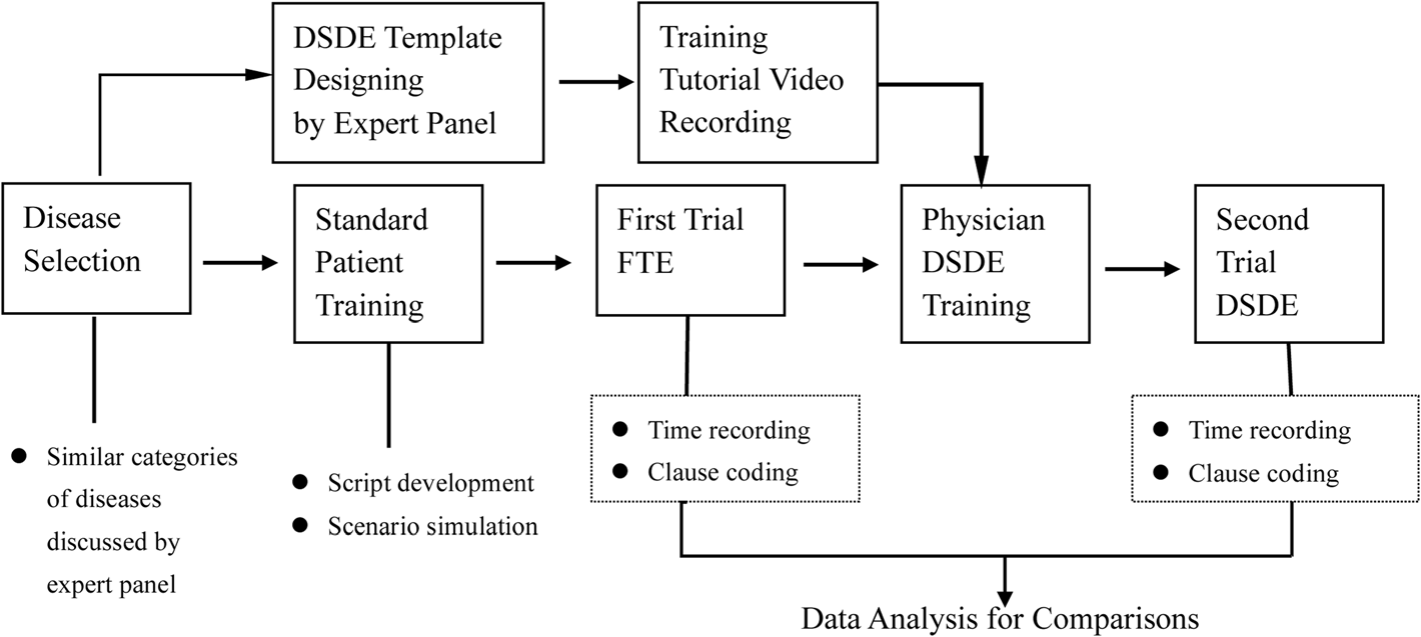
Flowchart of experiment design. *FTE* Free Trial Entry, *DSDE* Dynamic Structured Data Entry

The conceptual framework of the experiment appears in Fig. 2. An expert panel of three gynecologists in the department brainstormed a list of the conditions/clauses to include in the DSDE template. The results were reviewed by the panel coordinator and were subsequently reviewed and revised by physicians who served in the department, but not on the panel. This process took some 3 weeks. Once the final list of suggested template revisions was completed, it was reviewed by the original panel of three gynecologists, who determined the template’s final content. Physicians were trained to use the DSDE template via a 30-min tutorial video. Subsequent questions were raised and answered via direct dialogue between participants and the panel coordinator. Utilizing a single-grouped pretest and posttest design [18], we assessed physicians’ data entry output [19] using FTE and DSDE. Our quasi-experimental approach involved gathering data intensively on a small number of people, varying means of data entry (FTE and DSDE). Small sample sizes are quite common in research on the implementation of eMR, with many such studies involving very few physicians [20–23]. This method is particularly useful when participants’ time is scant and therefore extremely valuable. The within-subjects design, assessing output from each physician using both of the two data entry methods, controls effectively for individual-level noise in the data; that is, it controls for responses that are due to particular traits of the physicians themselves [24].

In this study, six female actors, each with a gynecological disease history, were recruited to have doctors’ visits at the hospital. Each female actor-patient followed a pre-designed script for possible physician questions customized to the woman’s actual, true medical history. The patient training materials were vetted by several experienced gynecologists. After attending a one-day training program prior to the experiment, the actor-patients were expected to behave like normal patients seeking a doctor’s opinion on / treatment for their actual disease. These procedures were designed to ensure that the patient-actors performances were consistent and reliable.

The actor-patients each had one of the following six gynecological diseases (International Classification of Diseases, or ICD 9 code): amenorrhea (626.0), dysmenorrhea (625.3), hyperprolactinemia (HPL) (253.1), premenstrual dysphoric disorder (PMDD) (625.4), polycystic ovarian syndrome (PCOS) (256.4), and premenstrual syndrome (PMS) (625.4). Each actor-patient presented with only one specific disease, regardless of ancillary conditions or diseases that she might have. These six diseases were selected because: 1) they are categorized similarly in the ICD 9, and thus share one template in the eMR system; 2) they are common in gynecology, and thus can be understood and assessed easily by physicians; and 3) these diseases can be unambiguously differentially diagnosed.

### Data collection

Data were collected from outpatient services in Taipei Veterans General Hospital (VGH). The focus of the investigation was on clinical notes recorded by physicians. Each female actor-patient visited each of the four participating gynecologists in random order for two separate trials, one for DSDE and another for FTE. Trial visits for each of the actor-patients were scheduled in a manner indistinguishable from those of regular patients in the gynecology department at VGH. The four physicians were chosen from the obstetrics and gynecology (OB/GYN) department at VGH and ranged in age from 35 to 55, with tenures at the hospital ranging from 7 to 27 years. With only a total of eight gynecologists employed in the OB/GYN department, our study involved 50% of the physician population for this specialty at the hospital. These participating physicians, all native-born Taiwanese, had similar educational backgrounds. None of them had prior experience in DSDE. Thus, the results of the study are not likely due to the particular features of the participants.

In the first trial, data entry into the eMR took place using the FTE interface, which had been developed by the in-house information technology team of VGH and is indeed the current system in use at VGH. In the second trial, data entry into eMR took place using DSDE, an interface adapted from the OpenSDE™ system that has been introduced since 2004 [4, 25]. The two trials were performed two months apart. As the physicians see hundreds of patients each week, the chance of the physicians recollecting the actor-patients from the initial meeting was quite minimal.

After the experiment, two measurements were collected: 1) the time spent by each physician preparing the medical record; and 2) the number of useful clauses included in the MR. The entire experimental process was video-recorded to precisely measure the length of time that each physician took to prepare each MR.

### Outcome measures

To objectively measure the quality of the medical notes written by the physicians, the output of the eMR preparation was evaluated on the dimensions of productivity, efficiency, and time-savings. Productivity was operationalized as the total number of meaningful clauses generated from each visit. A meaningful clause was defined as one that contained medical-related language including descriptions of patients’ feelings, behaviors, states of mind or conditions, terminologies, and so on. As patients are often transferred and/or referred to other doctors, medical notes provide a means for doctors to share information about the patients’ clinical history. More abundant information in the notes increases subsequent doctors’ assurance and peace of mind when treating the patients. Thus, the greater the number of clauses, the more productive the entry system. Efficiency was operationalized as the time needed for each clause entry (more specifically, the time-to-clause ratio). The less time needed for a clause entry, the more efficient. Outcome measures such as time savings and efficiency have been used by previous authors [7–9]. Previous research has operationalized productivity as the number of patients served, the number of diagnoses made, or the number of tests ordered [26]. Few, if any authors have directly counted the number of clauses as a measure of the amount and detail (hereafter, richness) of the medical notes.

The contents of the FTE eMR were coded for the number of concept clauses. The clause-based index constitutes a relatively novel method to measure the richness in medical records, especially for the FTE system. Physicians use clauses to express a concept in place of a full and complex sentence to improve writing efficiency. For example, “45 years old, RLQ pain, and FSH 38 mIU/ml”, would represent a 45-year-old woman with right lower-quarter pain and a blood level of follicle stimulating hormone of 38 mIU/ml; in our coding, this example would be considered to have three clauses. Essentially, each piece of information, including personal background, medical history, current symptoms, and disease condition was coded as a clause. It is important to note that physicians’ subjective descriptions, as well as objective findings, were analyzed and counted.

### Statistical methods

Differences between the two data entry systems’ outcomes were assessed using a paired t-test, as is appropriate for normally distributed data. Using the Shapiro–Wilk and Shapiro–Francia tests for normality, our outcome measures were assessed with the null hypothesis that data come from a normally distributed population [27, 28]. These methods have been widely used in studies with experimental designs similar to this one [18].

## Results

At the end of the experiment, a total of 48 (6x4x2) observations were collected; 24 for each trial (four physicians times six patients). The results appear in Tables 1, 2, 3. Specifically, Table 1 presents the length of time needed to prepare each medical note by physician by actor-patient. The length of time to prepare notes with DSDE was shorter than with FTE. Table 2 presents the number of clauses recorded for each medical note as a measure of the productivity of the eMR entry method. Table 3 presents the efficiency of recording medical notes by physician by patient. Efficiency was defined as the ratio of time spent recording-to-clause, such that the shorter the time needed to generate clauses, the greater the efficiency.

**Table 1:** Time length (minutes) for each medical record preparation

Disease	Physician A		Physician B		Physician C		Physician D	
	FTE	DSDE	FTE	DSDE	FTE	DSDE	FTE	DSDE
Amenorrhea	81	30	83	29	72	19	46	18
Dysmenorrhea	104	31	93	37	67	25	28	20
HPL	95	27	69	35	64	37	77	17
PMDD	101	24	62	28	73	33	48	14
PCOS	92	37	123	34	109	34	57	12
Premenstrual Syndrome	102	21	57	27	95	38	62	14
Total	575	170	487	190	480	186	318	95

**Table 2:** Productivity - Number of clauses recorded for each medical note preparation

Disease	Physician A		Physician B		Physician C		Physician D	
	FTE	DSDE	FTE	DSDE	FTE	DSDE	FTE	DSDE
Amenorrhea	6	11	7	10	5	5	5	9
Dysmenorrhea	11	12	6	17	7	10	6	13
HPL	11	17	11	13	9	12	7	11
PMDD	11	16	9	19	5	19	5	18
PCOS	11	15	7	15	9	13	5	10
Premenstrual Syndrome	11	16	8	17	6	14	7	14
Total	61	87	48	91	41	73	35	75

**Table 3:** Efficiency - Ratio of time-to-clause

Disease	Physician A		Physician B		Physician C		Physician D	
	FTE	DSDE	FTE	DSDE	FTE	DSDE	FTE	DSDE
Amenorrhea	13.50	2.73	11.86	2.90	14.40	3.80	9.20	2.00
Dysmenorrhea	9.45	2.58	15.50	2.18	9.57	2.50	4.67	1.54
HPL	8.64	1.59	6.27	2.69	7.11	3.08	11.00	1.55
PMDD	9.18	1.50	6.89	1.47	14.60	1.74	9.60	0.78
PCOS	8.36	2.47	17.57	2.27	12.11	2.62	11.40	1.20
Premenstrual Syndrome	9.27	1.31	7.13	1.59	15.83	2.71	8.86	1.00
Total	58.4	12.18	65.22	13.1	73.62	16.45	54.73	8.07

**Fig. 3:**
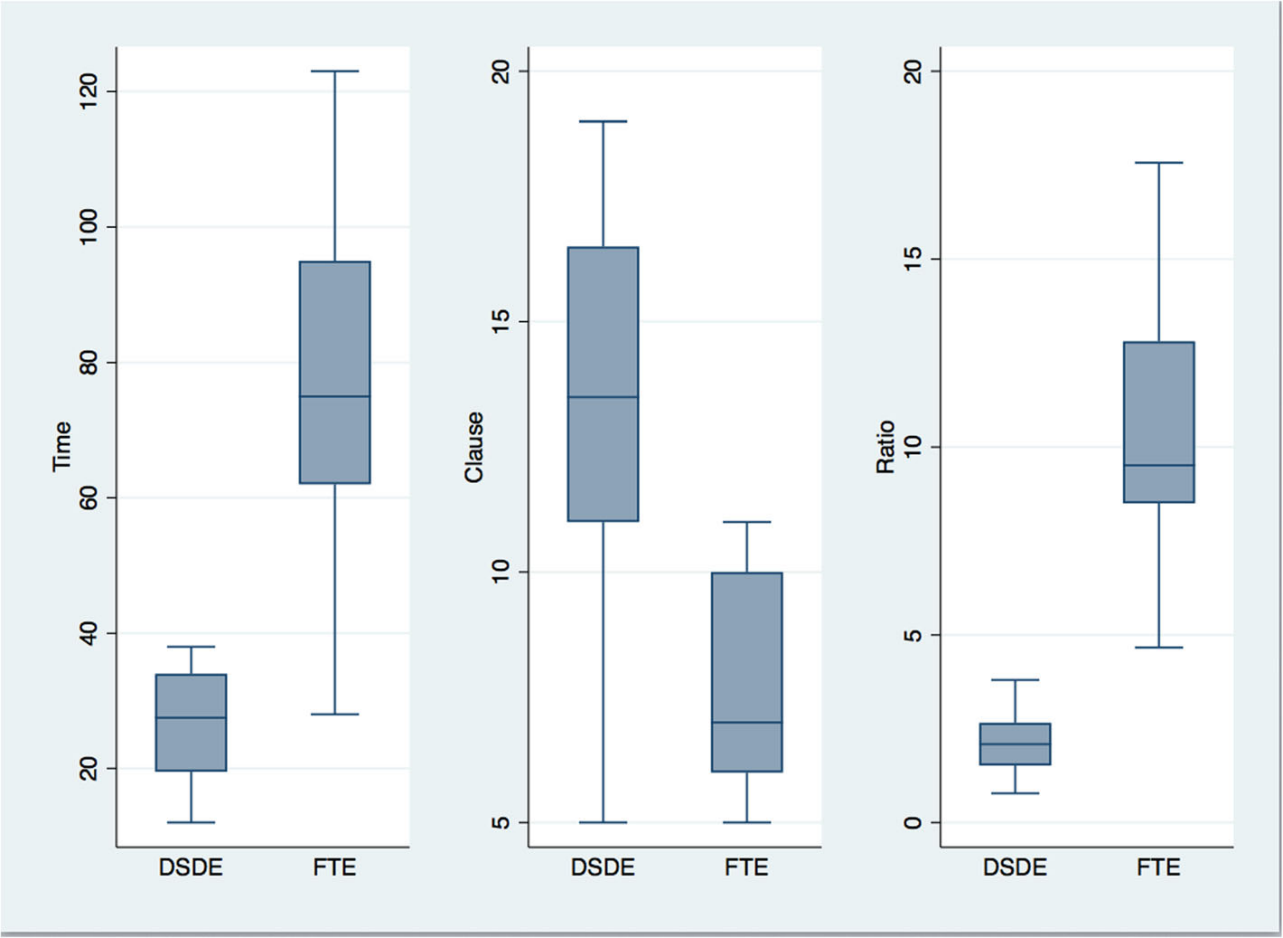
Outcomes by entry system. Note: The vertical axis of Time is measured in minutes describing the length of time that takes to complete a medical note. The vertical axis of Clause is the total number of meaningful clauses generated in each medical note. The greater the number, the more productive the entry system is. The Ratio is an efficiency measure that represents minutes needed for each clause entry; the shorter the time spent, the more efficient

**Fig. 4:**
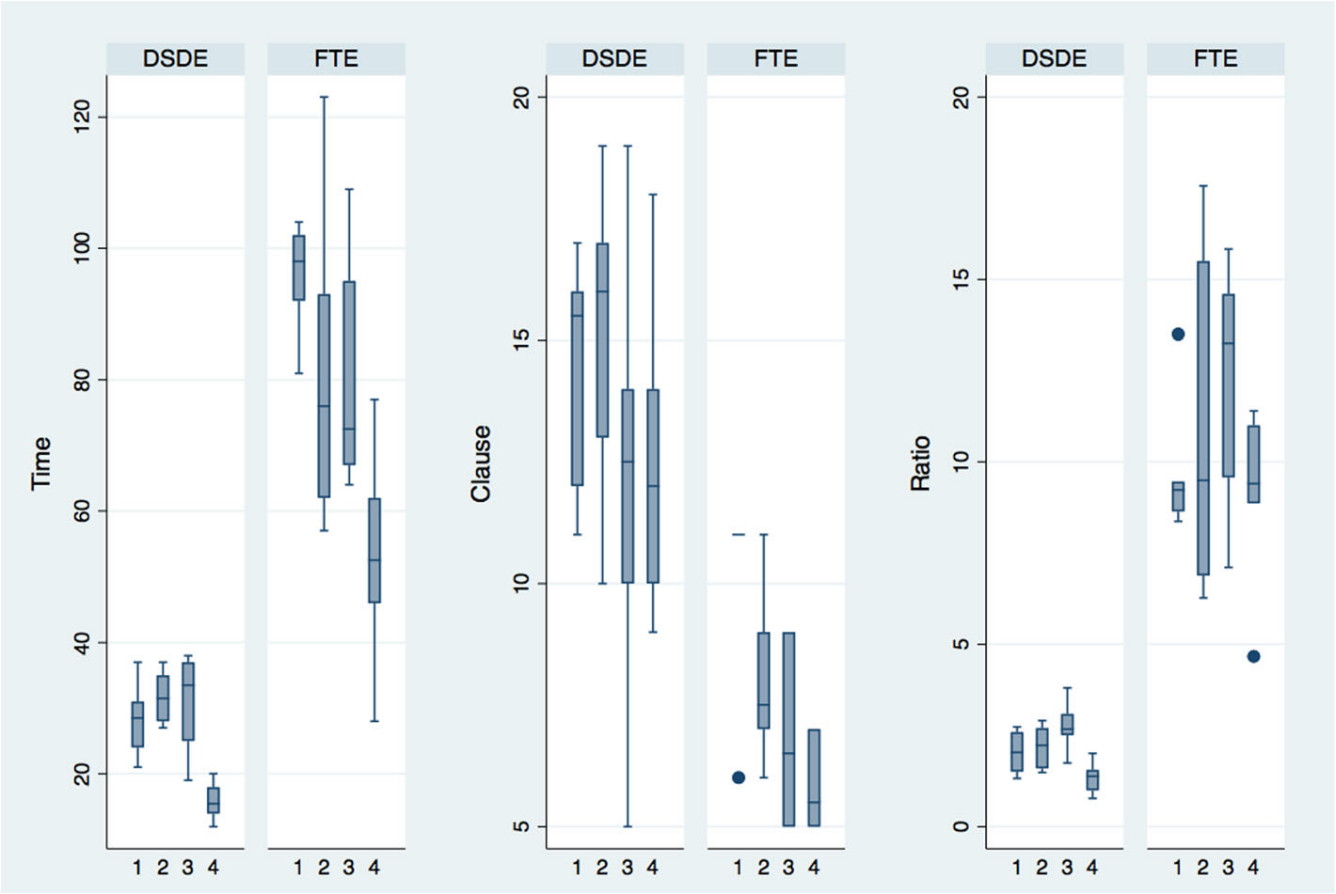
Outcome by entry system and physician. Note: The X-axis numbers represent individual physicians. The vertical axis is the same as that described in Fig. 3

**Fig. 5:**
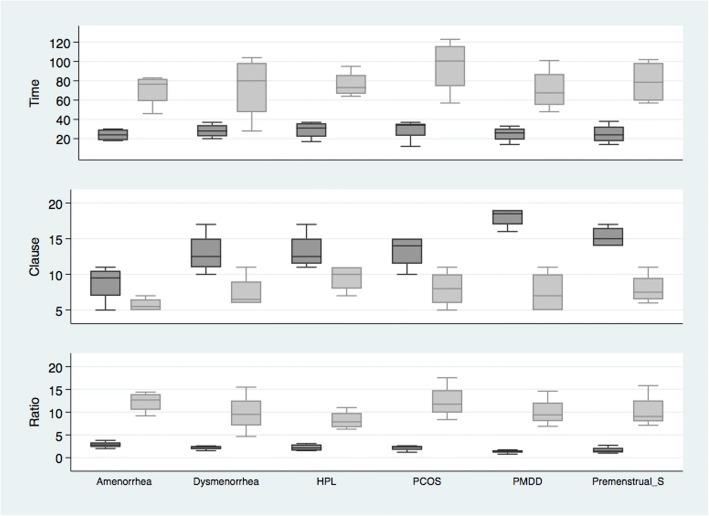
Outcome by entry system and diseases. Note: The X-axis lists the category of the six gynecological diseases represented by the six female actors. From left to right, they are Amenorrhea, Dysmenorrhea, HPL, PMDD, PCOS, and Premenstrual Syndrome. The vertical axis is the same as that described as in Fig. 2. The darker boxes represent DSDE and the lighter FTE

Graphical presentations of the results appear in Figs. 3, 4, 5. Figure 3, which graphically depicts the means from Table 5, shows that the total time needed (Time) to prepare each patient’s MR was substantially longer using the FTE system relative to the DSDE. Moreover, a greater number of clauses (Clause) were recorded using DSDE than FTE, and the time needed for each clause entry (Ratio) was shorter. These results hold true when the data are delineated by physician and disease, as shown in Figs. 4 and 5, respectively. Figure 4, displays mean values for each physicians’ results. For all three measures, Time, Clauses, and Ratio, the results from DSDE outperform those from FTE across physician. Figure 5 displays results by the 6 diseases assessed. The results of DSDE consistently outperform those of FTE across disease. These two figures suggest that the results are consistent across different diseases and physicians, and that the results are not a function of the particularities of either individual physicians or diseases.

Applying the Shapiro–Wilk and Shapiro–Francia tests, the results, featured in Table 4, indicate that the data for time, number of clauses (clause), and the time-to-clause ratio (ratio) were normally distributed for both FTE and DSDE. Thus, a paired t-test was used to assess the differences between DSDE and FTE for productivity, efficiency, and time-saving.

**Table 4:** Normality tests

		Shapiro-Wilk W test		Shapiro-Francia W′ test	
		*W*	*p*	*W′*	*p*
Time	DSDE	0.96	0.17	0.94	0.38
	FTE	0.99	0.98	0.99	0.96
Clause	DSDE	0.98	0.64	0.97	0.88
	FTE	0.97	0.60	0.97	0.48
Time-to-clause	DSDE	0.96	0.50	0.96	0.45
	FTE	0.96	0.40	0.96	0.41

*Note:* All results suggest that the null hypothesis should not be rejected and that these variables were normally distributed

The test results, shown in Table 5, indicate that DSDE and FTE were significantly different for all three outcomes. Specifically, DSDE shows an average of 97% fewer minutes needed and 55% more clauses recorded for each eMR compared to FTE. In addition, for each clause entry in a medical note, the time saved was 133%. With results in Table 5 significant at the 1% level, the data suggest that DSDE not only saves eMR preparation time, but also enriches the contents of the eMR relative to FTE. The findings suggest that eMR systems can be much more productive and efficient when used with DSDE.

**Table 5:** Paired t-test results

		Mean	S.D.	Diff (%)	*t*	*p*
Time	DSDE	26.71	8.22	−97.48	−12.55***	.00
	FTE	77.50	22.91			
Clause	DSDE	13.58	3.50	55.14	8.01***	.00
	FTE	7.71	2.29			
Time-to-clause	DSDE	2.08	.75	−133.86	−12.99***	.00
	FTE	10.50	3.34			

*Note:* The mid-point method was used for calculating percentage changes for the means

## Discussion

Our study made use of hospital outpatient visit trials to produce eMR comparisons through a quasi-experimental design. Although the sample size was relatively small, the study is based upon actual visits between doctors and patients. Twenty-four samples were collected for FTE and DSDE each. With a careful research strategy, the relatively small number of cases generated significant results supporting the assertion that DSDE provides more medical information per time spent entering than FTE.

The time efficiency and time saving generated by DSDE is especially important for a non-English-speaking country, such as Taiwan. Though it is customary for physicians to record medical notes and write orders in English; writing English-based eMR in FTE can still be challenging. Thus, this DSDE system might significantly reduce language-based errors and support physicians in constructing English-based medical notes by “pointing and clicking” on pre-designed phrases and clauses.

Using female actors as representative patients in the experiment constitutes a novel innovation of our study. Most previous studies recruit volunteers or regular patients as participants [7, 9, 12, 14]. However, regular volunteers/patients making multiple visits to the doctor may generate biased results; there may be inconsistencies between the patients’ earlier and later visits as a result of learning, i.e., multiple treatment interference [29]. By contrast, the female actors were trained and indeed accustomed to acting naturally even as they repeated speeches many times over. Consequently, when speaking to four different physicians over the two trials, they were able to describe their disease conditions credibly and consistently with minimum variations over the course of the eight visits in which they participated. For this reason, female actors with similar disease backgrounds, instead of the more common volunteer patients, were used in the study to ensure and extend research validity.

Another novel feature of this research was the inclusion of outcome measures that assessed productivity, efficiency, and time saving of the eMR entry system. The extant literature focuses primarily on physicians’ satisfaction rates to demonstrate the superiority of the eMR system [3, 30–32]. This study identified the differences between the two eMR entry systems with more concrete and objective measures. EMR generated via FTE were analyzed using clause-counts, with each symptom or sign interpreted as a single clause. Previous research has assessed the richness of FTE-generated eMR using straight word counts. However, irrelevant or unintelligible content can be included in FTE-generated eMR potentially reducing the accuracy of the measure. Our study thus provides a more precise measure of the richness of FTE than straight word counts.

This study contributes to the literature by informing hospital administrative personnel about the degree to which DSDE systems improve the quality and quantity of eMR and enhance data-entry efficiency for clinicians relative to traditional free-text entry systems. The existing literature has primarily compared computer to paper data entry methods, and has found that computerized data entry methods result in anything from 22.2% time gained to 40.6% time lost [7, 12, 20]. From these findings, it is hoped that DSDE will be increasingly implemented for eMR in hospital outpatient settings to achieve reductions in the time needed to do entry as well as to increase the depth of content. Finally, it should be noted that free-form text and structured data entry are not mutually exclusive. For example, an eMR that uses a structured vocabulary could also feature a text box for free-form entry of clinical notes. Moreover, the level of detail that is appropriate in a clinical note may differ depending on who will be using the eMR and what data users want to search for and aggregate.

### Limitations

Our study possesses some limitations. All participating physicians were middle-aged or younger doctors, either rising professionally or at the peak of their career paths. Older clinicians, who may be uncomfortable in computerized settings, may take longer to be familiar and adept with new systems. In such cases, the transition costs could be higher than expected. All of the six selected diseases are categorized similarly in the ICD. The purpose of this selection was to ensure that clinicians can be easily familiarized with the template used in the DSDE form, and confusion during data entry could be minimized. In reality, however, when clinicians encounter patients with rarer diseases whose symptoms do not readily fit into DSDE, this system would have to be expanded and re-formulated to include new templates. The results for rare or more complicated diseases are, thus, beyond the scope of our study. Thirdly, our study was relatively modest, with a sample of only four physicians and six participating patients, all from the same hospital. However hard we have tried to minimize potential biases, the positive results of the study should be viewed with caution. The usual concerns surrounding small sample sizes hold for this study including the risk that results could be driven by a particular participant or the particular hospital in which the study was conducted; such concerns increase the likelihood of Type II error. Another limitation is the potential for an observer effect. The knowledge that the participants’ medical notes would be scrutinized by researchers, may have influenced aspects of the gynecologists note preparation. The increases in efficiency, productivity, and time saving demonstrated in our study may partly be a function of the increased ease with which non-English speaking physicians could “tick” boxes.

## Conclusions

This study employed a quasi-experimental design and demonstrated that DSDE has the potential to be more productive, time-saving, and efficient than FTE for clinicians in the documentation of outpatient medical records. Six female actors were recruited to participate in the experiment as representative patients with six gynecological diseases. The experimental design is novel and useful, especially in healthcare organization settings where observations are typically few and participants’ time extremely valuable. With its focus on the documentation outcomes of outpatient visits, the study provides evidence for the superiority of DSDE in eMR relative to FTE at the single visit level. However, due to the limitations of the small sample size, additional user studies are needed to verify the generalizability of these results. Further research on DSDE might make use of aggregate measures of the number of services provided, number of events prevented, or even overall cost benefit analysis as measures of efficiency and productivity; such research will provide an improved understanding of the implementation of this new technology.

## Abbreviations

CPOE: Computerized physician order entry
DSDE: Dynamic structured data entry
eMR: Electronic medical record
FTE: Free text entry
HPL: Hyperprolactinemia
ICD: International classification of disease
PCOS: Polycystic ovarian syndrome
PMDD: Premenstrual dysphoric disorder
VGH: Veterans general hospital
